# RNA-seq for comparative transcript profiling of kenaf under salinity stress

**DOI:** 10.1007/s10265-016-0898-9

**Published:** 2016-12-20

**Authors:** Hui Li, Defang Li, Anguo Chen, Huijuan Tang, Jianjun Li, Siqi Huang

**Affiliations:** Institute of Bast Fiber Crops, Chinese Academy of Agricultural Sciences, No. 348 West Xianjiahu Road, Changsha, 410205 China

**Keywords:** High-throughput sequencing, Kenaf, Salt tolerance, Transcriptome

## Abstract

Kenaf (*Hibiscus cannabinus* L.) is an economically important global natural fiber crop. As a consequence of the increased demand for food crops and the reduction of available arable land, kenaf cultivation has increasingly shifted to saline and alkaline land. To investigate the molecular mechanism of salinity tolerance in kenaf, we performed Illumina high-throughput RNA sequencing on shoot tips of kenaf and identified 71,318 unigenes, which were annotated using four different protein databases. In total, 2,384 differentially expressed genes (DEGs) were identified between the salt-stressed and the control plants, 1,702 of these transcripts were up-regulated and 683 transcripts were down-regulated. Thirty-seven transcripts belonging to 15 transcription-factor families that respond to salt stress were identified. Gene ontology function enrichment analysis revealed that the genes encoding antioxidant enzymes were up-regulated. The amino acid metabolism and carbohydrate metabolism pathways were highly enriched among these DEGs under salt stress conditions. In order to confirm the RNA-seq data, we randomly selected 20 unigenes for analysis using a quntitative real-time polymerase chain reaction. Our study not only provided the large-scale assessment of transcriptome resources of kenaf but also guidelines for understanding the mechanism underlying salt stress responses in kenaf.

**Electronic supplementary material:**

The online version of this article (doi:10.1007/s10265-016-0898-9) contains supplementary material, which is available to authorized users.

## Introduction

Soil salinization has limited the ability to respond to the increase in demand for food crops. Globally, more than 20% of the available agricultural land is affected by salinity, and this area is predicted to double by 2,050 (Hameed et al. 2012; Rengasamy 2006). The reduction of arable land and the shortage of freshwater resources will force the development of saline and alkaline land, coastal zone areas, and tidal flats. Therefore, the mechanism of salt tolerance has become a focus of research.

To improve the yield of crops under salt stress conditions, it is essential to understand the fundamental molecular mechanisms behind salinity tolerance. Salinity-stress tolerance is a quantitative trait that is controlled by multiple genes (Chinnusamy et al. 2005). Salinity tolerance involves a complex of responses at molecular, cellular, metabolic, physiological, and whole-plant levels. The regulation of gene expression under salinity stress includes transcriptional control, RNA processing, and protein modification. Next-generation sequencing (NGS) has been used to determine the diversity of salt-stress responses on a transcriptome-wide scale in non-model plant species, where the whole genome sequence and annotation are not yet available. Comparative transcription analysis can provide detailed information about a broad spectrum of genes that respond to salt stress, including salt-responsive transcription factors, and up- or down-regulated genes. NGS is widely used to screen candidate and clone genes involved in salt-stress responses. An extensive number of salt-responsive genes have been identified and characterized such as *dreb1*/*cbf, dreb2, and areb*/*abf* (Choudhury et al. 2008; Fujita et al. 2011, 2013; Ito et al. 2006; Mizoi et al. 2012).

Kenaf (*Hibiscus cannabinus* L.) is one of the most important fiber crops, which has multiple uses, including paper pulp, carpet backing, building materials, and livestock forage(Alexopoulous et al. 2000; Banñuelos et al. 2002; Kemble et al. 2002; Webber et al. 2002). It is currently cultivated in more than 20 countries, predominantly in China, India, and Thailand (Liu 2000). With the reduction of agricultural land, increased populations, and the demand for food crops, kenaf cultivation has been relocated to saline and alkaline land. Therefore, elucidation of the mechanism of salinity tolerance and improvement of resistance to salt stress are important for the development of the kenaf industry.

Recently, research on kenaf salt tolerance has made some progress. It was found that sodium salt solutions (Na_2_SO_4_ and Na_2_CO_3_) had different effects on kenaf seed germination and seedling growth. When a 50 mmol/l Na_2_SO_4_ solution was applied to kenaf seeds, the germination rate was not significantly different from the control, whereas a 50 mmol/l Na_2_CO_3_ solution considerably inhibited seed germination rates (Liu and Li 2011). Salt stress significantly inhibited the root and seedling growth of kenaf, and the responses of different genotypes to salt stress were different. An F1 hybrid of kenaf showed positive mid-parent and over-parent heterosis within a certain salt concentration range (Zhang et al. 2011), suggesting strong heterosis with salt tolerance. Therefore, we can use hybrid breeding to improve the salt tolerance of kenaf. In response to salinity stress, plants develop various physiological and biochemical mechanisms to survive, such as antioxidant enzyme activation and antioxidant compound synthesis.

However, a genome-wide analysis of gene expression patterns in response to salt stress has not been undertaken in kenaf. In the present study, we performed a genome-wide analysis of gene expression in Zhonghongma variety 16 under salt and non-salt stress conditions using Illumina next-generation sequencing. Through a comparative transcriptome analysis, we identified several salt-stress responsive genes encoding salt-responsive transcription factors, biosynthesis of compatible solutes, and antioxidant enzymes. To the best of our knowledge, this is the first report to provide molecular insights into the salinity tolerance of kenaf.

## Materials and methods

### Plant material

As different genotypes of kenaf respond differently to salt stress, we selected 100 varieties of kenaf to determine which variety showed the strongest resistance to NaCl stress. From the 100 varieties, we selected 20 with strong resistance to salt stress conditions. Seeds of the 20 varieties were planted in a Dafeng saline and alkaline test plot to further test their salt resistance. Zhonghongma 16 was found to be one of the strongest salt-stress resistant kenaf varieties, so it was selected as the experimental variety.

Seeds of Zhonghongma 16 were washed with de-ionized water and planted on nursery seeding plates. After 10 d, we selected seedlings showing strong and consistent growth, and transferred them to hydroponic systems with half-strength solution and air bubbling for further growth (25 ± 2 °C, 12 h light and dark cycle) in a plant growth room. After 5 days, the seedlings were divided into two groups: No. 1 were grown under normal conditions; No. 2 were grown in a concentration of 250 mM NaCl. After 2 weeks, we collected 10 shoot tips from each of three biological replicates of each group, the shoot tips were frozen in liquid nitrogen and stored at −80 °C until use. The No. 1 group seedlings were used as the control sample (CK), and the three biological replicates were named as CK1, CK2, and CK3; the No. 2 group seedlings were used as the salinity-stress sample (ST), and the three biological replicates were named as ST1, ST2, and ST3.

### RNA extraction and cDNA library preparation and sequencing

The shoot tips from each group were powdered in liquid nitrogen using a mortar and pestle and the total RNA was extracted using TRIzol reagent according to the manufacturer’s protocol (Invitrogen, Camarillo, CA, USA). The quality of the total RNA was verified using an Agilent 2100 Bioanalyzer before further processing. Each RNA fraction used to prepare a separate cDNA library were CK1, CK2, CK3, ST1, ST2, and ST3 for sequencing and the sequencing was performed at the Beijing Genomics Institute (BGI; Shenzhen, China). Poly-(A) mRNA was isolated from total RNA with Magnetic Oligo (dT) Beads. First, the purified mRNA was fragmented into small pieces, and then double-stranded cDNA was synthesized using the SuperScript Double-Stranded cDNA Synthesis kit (Invitrogen) with a random hexamer (N6) primer (Illumina). The double-stranded cDNA was subjected to end-repair and phosphorylation using T4 DNA polymerase, Klenow DNA polymerase, and T4 polynucleotide kinase. These repaired cDNA fragments were 3′-adenylated using Klenow 3′–5′ exo-polymerase, then ligated with Illumina paired-end adapters to the end of these fragments using T4 DNA ligase. The adapter-ligated fragments were separated on an agarose gel and cDNA fragments of approximately 200 bp (±25 bp) were excised from the gel. To enrich the purified cDNA template, The PCR was performed to amplify the cDNA fragments. The amplification protocol comprised a 30 s incubation at 98 °C then a cycle of 98 °C for 10 s, 60 °C for 30 s, 72 °C for 30 s, repeat 14 times, 72 °C for 5 min, and a final 10 °C hold. The cDNA library was constructed with a fragment length of 200 bp (±25 bp). After validation with an Agilent 2100 Bioanalyzer, the cDNA library was sequenced on a PE flow cell using an Illumina Hiseq2000 sequencing platform.

### Data filtering and assembly

Before assembly of the kenaf transcriptome, a stringent filtering process was carried out. The adapter sequences of the raw reads were removed, and low-quality sequences (reads with ambiguous bases ‘N’) and reads with more than 20% Q < 20 bases were removed. Assembly of the clean reads was performed using Trinity software. The Trinity software was used to combine reads with overlapping nucleic acid sequences to form contigs. Subsequently, contigs from the same transcript were assembled and sequences that could not be extended at either end were defined as unigenes. TGICL software was used to eliminate redundant unigenes and to further assemble unigenes to form a single non-redundant set. Unigenes from all libraries were assembled again to acquire non-redundant unigenes (All-Unigene set) that were as long as possible.

### Functional annotation and classification of assembled unigenes

All the assembled high-quality unigenes were first screened by BLASTX (E-value cut-off of 10^− 5^) against the National Centre for Biotechnology Information (NCBI) non-redundant (NR) database. Additionally, the all-unigenes were aligned to the other five public databases, including NCBI nucleotide sequences (Nt), Swiss-Prot, KEEG, COG, and GO databases. Based on NR annotation we used the Blast2GO program (Conesa et al. 2005) to obtain GO annotation of unigenes, and WEGO software (Ye et al. 2006) for GO functional classification of all-unigenes; these analyses provided information on the distribution of kenaf gene functions. Unigene sequences were also aligned to the COG database to predict possible functions, and to determine the gene function distribution characteristics of kenaf. The KEGG database was used to analyze the gene product during the metabolism process and related gene function in cellular processes (Kanehisa and Goto 2000).

### Differentially expressed genes analysis

To identify the differentially expressed genes (DEGs) between the CK and ST groups, the expression levels of all the transcripts analyses were determined using DESeq (Anders and Huber 2010). Unigenes were identified as differentially expressed when FDR ≤ 0.001 and the absolute value of log2 ratio ≥ 1 was set as the threshold.

### Quantitative RT-PCR analysis

The total RNA was extracted using TRIzol reagent according to the manufacturer’s protocol (Invitrogen, Camarillo, CA, USA) from CK and ST samples. To evaluate the validity of comparative transcriptome analysis of the patterns of DEGs, 20 candidate unigenes (14 up- and 6 down-regulated) were selected and validated by quantitative real time PCR (qRT-PCR).

qRT-PCR was performed using SYBR Green qPCR Master Mix (Takara) on a iQ5™ multicolor real-time PCR detection system (Bio-Rad). The volume of the qRT-PCR reaction was 10 µl, containing 2 µl cDNA, 5 µl 2 × SYBR Green qPCR Master Mix, 0.3 µl of the forward and reverse primers, and 2.4 µl ddH_2_O. Actin was used as the endogenous reference gene. The reactions were incubated at 95 °C for 2 min, followed by 40 cycles of 95 °C for 15 s, 61 °C for 15 s, and 72 °C for 20 s. After the PCR, a melting curve was generated by gradually increasing the temperature to 95 °C to test the amplicon specificity. To determine relative fold differences for each sample, the CT value for each gene was normalized to the CT value for the reference gene and was calculated relative to a calibrator using the DDCT method as described by Livak and Schmittgen (2001). All reactions were run in three replicates for each sample. The primer pairs used for qRT-PCR are shown in Table S1.

## Results

### Illumina paired-end sequencing and assembly

To obtain a global overview of the kenaf gene expression profile and the salt stress genes, total RNAs were extracted from shoot tips of CK and ST plants. To minimize bias from the Illumina sequencing and transcriptome sampling, we constructed 6 cDNA libraries: CK1, CK2, CK3, ST1, ST2, ST3, and sequenced these separately using an Illumina Hiseq2000 genome analyzer.

Using the Illumina Hiseq2000 sequencing platform, each sequenced sample yielded 2 × 90 bp independent reads from either end of a cDNA fragment, approximately 159.5 million (CK) and 160.9 million (ST) raw reads were generated. After a stringent quality assessment, 146.4 million (CK), 147.7 million (ST) clean reads were obtained with 97.77% Q20 bases (Table 1).

**Table 1:** Output statistics of sequencing

Samples	Total raw reads	Total clean reads	Q20%	GC %
CK1	52,922,062	48,326,998	97.70	46.05
CK2	54,691,954	50,382,074	97.76	45.47
CK3	51,859,144	47,656,960	97.77	45.42
ST1	53,887,700	49,358,176	97.77	45.48
ST2	51,881,472	47,594,544	97.77	45.51
ST3	55,145,768	50,714,614	97.74	45.21

Total reads are clean reads; Q20% is the proportion of the nucleotide quality value larger than 20; GC % is proportion of guanidine and cytosine nucleotides among the total nucleotides

The data were deposited in the NCBI Sequencing Read Archive database (accession number SRP064405). The high quality reads were assembled into one reference transcriptome using the Trinity software.

**Fig. 1:**
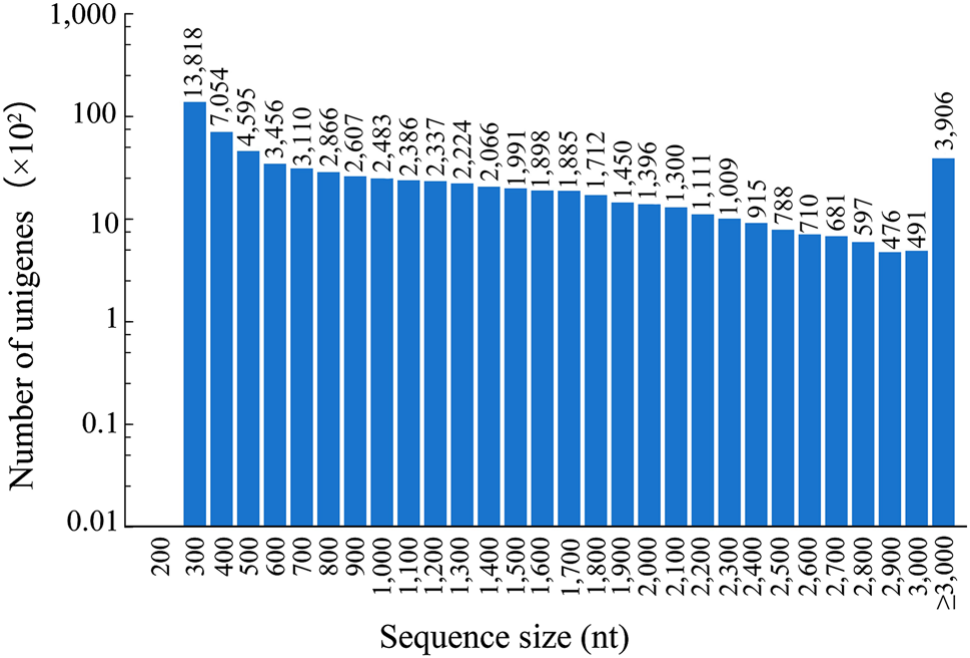
The length distribution of all unigenes

Using the Trinity assembler, 71,318 unigenes were generated with an average length of 1,143 nt, an N50 of 1,784 nt, and a total length of 81,509,2256 nt. The total unigenes included 13,818 (19.38%) of less than 300 nt, 54,085 (75.84%) with lengths from 301 to 3,000 nt, and 3,415 (4.78%) with lengths greater than 3,000 nt (Fig. 1).

### Functional annotation and analysis

The annotation of the 71,318 assembled unigenes revealed that 56,147 (78.72%), 38,065 (53.37%), 33,807 (47.40%), 22,049 (30.92%), and 51,851 (72.7%) showed significant similarity to known proteins in the Nr, Swiss-Prot, KEGG, COG, and Nt databases, respectively. In total 58,095 unigenes (81.46% of the total unigenes) were successfully annotated in at least one of the above databases (Table 2.)

**Table 2:** Statistics of annotation results

Sequence file	NR	NT	Swiss-Prot	KEGG	COG	GO	ALL
All unigenes	56,147	51,851	38,065	33,807	22,049	45,855	58,095

All unigenes were aligned to the COG and GO databases for functional prediction and classification. The Gene Ontology (GO) database has three principal categories: biological process, cellular component, and molecular function. On the basis of Nr annotation, 45,855 unigenes were assigned to these three categories, and were distributed across 45 functional categories. For the biological process, 65.29% were cellular process unigenes, and 61.32% were metabolic process unigenes. For the cellular component, 77.62% of the unigenes were assigned to cells and 77.6% to cell parts.

**Fig. 2:**
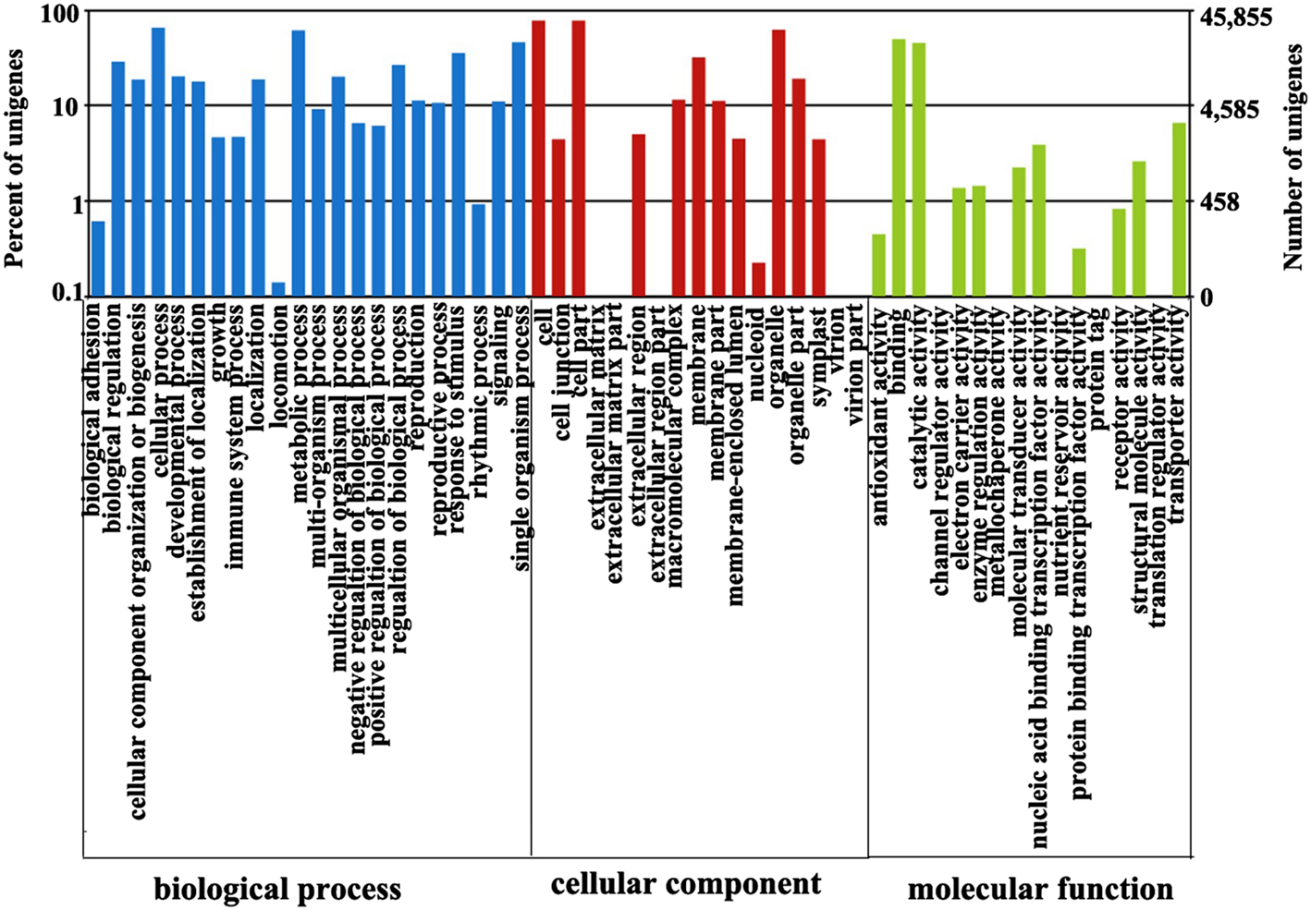
Gene ontology (GO) classifications of all unigenes. The results are summarized in three main categories: biological processes, cellular components, and molecular functions

The GO classifications of all unigenes are shown in Fig. 2.

**Fig. 3:**
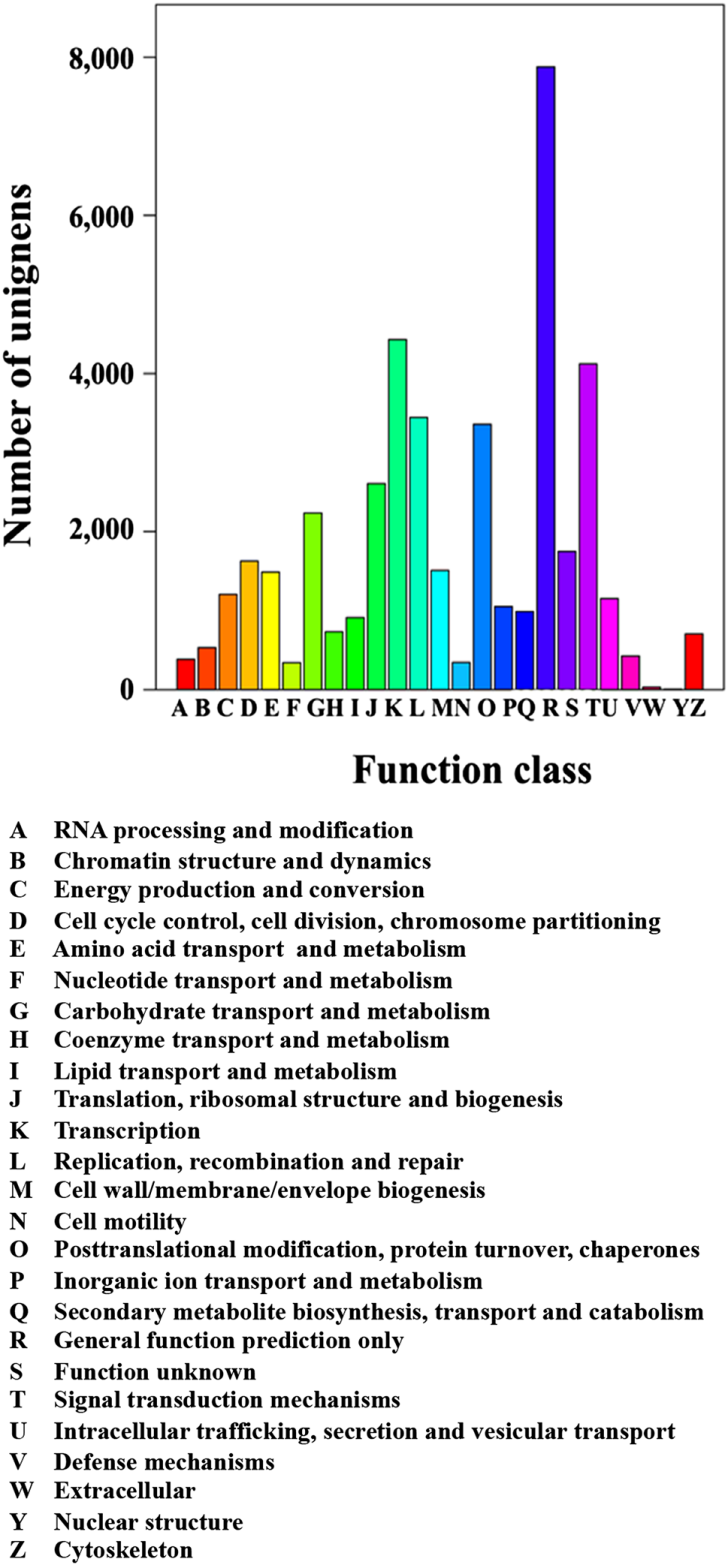
Clusters of Orthologous Groups (COG) classification of all unigenes

The COG database was used to classify orthologous gene products. In total, 22,049 unigenes were assigned to the 25 COG categories. Of the 25 COG categories, the most frequently identified classes were general function (35.7%), transcription (20.1%), and signal transduction mechanisms (18.7%); a few unigenes were assigned to the nuclear and extracellular structure categories (Fig. 3).

The KEGG Pathway database records the networks of molecular interactions in cells and species-specific variations. Pathway-based analysis provides insight into the biological functions and interactions of genes. Using BLASTX with an E-value threshold of 10^− 5^, 33,807 unigene sequences were grouped into 128 KEGG pathways, which are shown in Table S2.

### Analysis of differential expression of assembled kenaf transcripts under salt stress

**Fig. 4:**
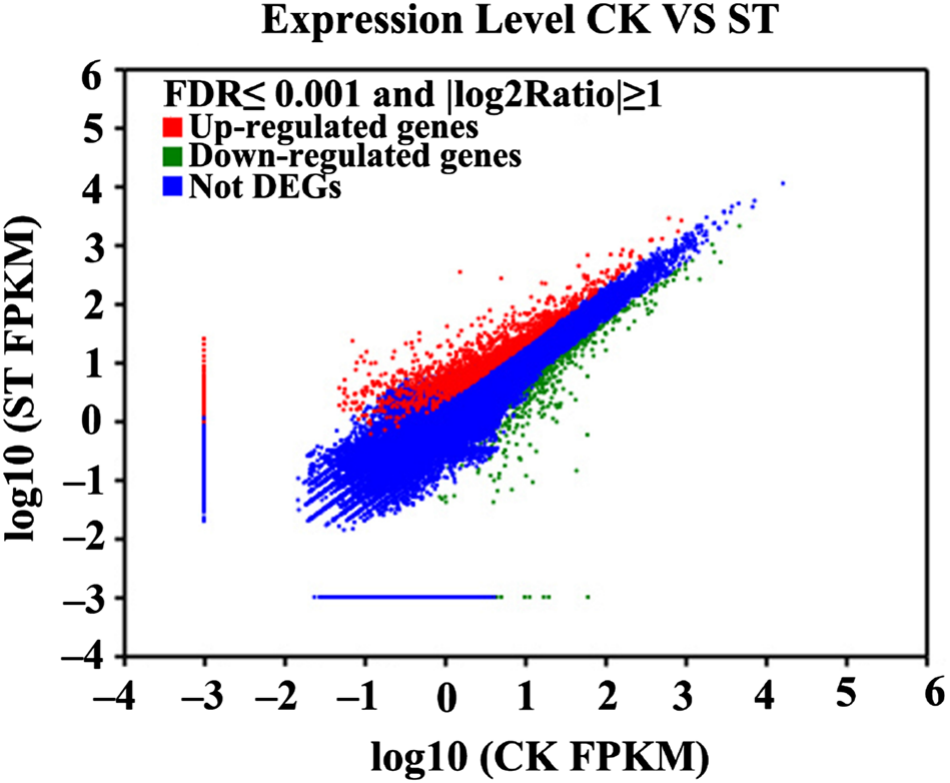
Differentially expressed genes

Compared with the crontrol, 2,385 contigs were classified as differentially expressed genes(DEGs) in salt-treated kenaf, among these DEGs, 1,702 transcripts were up-regulated and 683 transcripts were down-regulated (Fig. 4).

GO annotation and statistical analyses demonstrated that 1,789 tested DEGs (some contigs had more than one GO annotation) were classified in three GO ontologies and 49 terms. Among these GO categories, cell, cell part and metabolic process were significantly enriched among DEGs compared to the whole transcriptome background. In the molecular-function category, 971 tested DEGs which were up-regulated (some contigs had more than one GO annotation) were enriched in 581 terms. The overwhelming majority of the genes were related to “binding” (GO: 0005488,566 DEGs) (Table S3). Through a GO function enrichment analysis, we found that the genes coding antioxidant enzymes were up-regulated, such as those for peroxidase, glutathione transferase, and superoxide dismutase (Table S4).

In our analysis, 2,384 tested DEGs mapped to 117 pathways with most mapping to “metabolic pathways” (501 DEGs), “biosynthesis of secondary metabolites” (302 DEGs), and “plant hormone signal transduction” (90 DEGs) (Table S5). Through KEGG enrichment analysis, we found that some unigenes in amino acid metabolism pathway and carbohydrate metabolism pathway were up-regulated (Table S6).

### Transcription factors responding to salt stress expression

We performed a global transcription-factor classification for differentially expressed transcripts and identified 37 transcripts belonging to 15 transcription-factor families (Table S7). Among them, 21 transcription-factors were up regulated, and 14 transcription-factors were down regulated. AP2/ERF domain-containing transcription factor was strongly upregulated under salinity treatment. Of the eight WRKY-family members, more were downregulated under salt treatment.

### Verification of RNA-Seq data by qRT-PCR

To evaluate the validity of Solexa analysis of the patterns of DEGs, we selected 20 candidate unigenes (14 up- and 6 down-regulated), which responded to salt stress and were detected by qRT-PCR. Solexa sequencing revealed that the expression patterns of 17 out of 20 (85%) unigenes showed general agreement with Solexa sequencing. However, the patterns of arginine decarboxylase, gibberellin-regulated protein, and photosystem I reaction center V unigenes did not agree with the Solexa sequencing. They were detected as identically expressed in the CK and ST plants by qRT-PCR. The confirmation of the expression profiles of selected genes by qRT-PCR is shown in Table 3.

**Table 3:** Confirmation of the expression profiles of selected genes by qRT-PCR

Unigene	Protein identity	Log2 ratio	qRT-PCR
CL2927.Contig1	Beta-amylase 3	13.04	21.61
CL554.Contig12	Class III HD-Zip protein	11.97	7.01
Unigene27652	Sodium/hydrogen exchanger	11.63	12.11
CL3883.Contig4	WRKY transcription factor 28	9.457	12.71
Unigene13798	Zinc finger protein	5.133	1.21
CL3599.Contig1	Trehalose synthase	4.952	6.57
CL541.Contig2	Trehalose-6-phosphate synthase	3.128	10.39
CL9910.Contig3	Pyrophosphate-energized vacuolar membrane proton pump	2.936	2.41
CL6853.Contig2	ABC transporter C family	2.367	6.85
CL897.Contig4	bZIP transcription factor	2.339	7.65
Unigene10019	ATP-dependent zinc metalloprotease	2.162	8.14
Unigene9404	Plasma membrane Na+/H + antiporter	1.870	3.70
Unigene7141	Galactinol—sucrosegalactosyltransferase	1.687	2.86
CL184.Contig22	Arginine decarboxylase	1.470	0.71
Unigene28843	Cytochrome P450	−9.00	0.77
Unigene1983	Photosystem I reaction center V	−4.07	3.58
Unigene17637	Chlorophyll a/b-binding protein	−3.56	0.85
Unigene9851	Gibberellin-regulated protein	−2.52	5.78
CL5512.Contig1	Light-harvesting complex II	−2.03	0.87
CL8228.Contig1	Sugar transport protein	−1.98	0.33

## Discussion

With the development of sequencing technology, NGS has enabled a novel gene discovery on a genome-wide scale. As NGS is low cost, with high efficiency and accuracy, it has been widely used to sequence transcriptomes and has been successfully applied to de novo transcriptome sequencing and assembly in many plants, e.g., bamboo, rubber, and ramie (Li et al. 2012; Liu et al. 2012, 2013).

In the present study, using of NGS and Illumina paired-end sequencing technology enabled the characterization of the kenaf transcriptome and the assembly of 71,318 unigenes. Approximately 78.72% of the unigenes had homologs in the Nr protein database, and their functions could be annotated by searches of public databases. These functions were also classified by COG and GO and the metabolic pathways were ascertained using the KEGG database.

Transcriptome analysis provides detailed knowledge about the gene expression at the mRNA level, which is widely used to screen candidate genes involved in stress responses. Through comparative transcriptome analysis, we revealed some genes involved in salinity stress response including transcription factors, and enzymes involved in the biosynthesis of compatible solutes that were generated during the osmotic phase. These results will aid in the elucidation of the molecular mechanisms of salinity tolerance in kenaf.

### Antioxidant defence of salinity tolerance

Salinity stress is considered as hyperosmotic stress, which limits plants growth and yield. Osmotic stress involves changes in various physiological and metabolic process such as denaturation of proteins interruption of cell membrance nutrient imbalance, which giving rise to the accumulation reactive oxygen species (ROS) (Rahnama et al. 2010; Munns and Tester 2008).

An excess of ROS is harmful to the plants and consequently, they have evolved strategies to detoxify the ROS by the up regulation of antioxidants. The antioxidant enzymes comprise several enzymes such as peroxidase activity (POD), glutathione transferase, superoxide dismutase (SOD), catalase (CAT), glutathione reductase (GR). Under salt-stress conditions in kenaf, catalase and glutathione reductase activity increased with increasing salt concentrations (Chen et al. 2011).

In the present study, GO function analysis revealed that activities of peroxidase, glutathione transferase, and superoxide dismutase were up regulated under salinity stress. The results showed that antioxidant enzymes play a critical role in detoxifying ROS induced by salt stress.

### Compatible osmolytes of salinity tolerance

Compatible osmolytes such as proline, soluble sugar, free amino acids, and polyols (Hossain et al. 2011; Tahir et al. 2012; Nounjan et al. 2012) constitute a group of chemically diverse organic compounds that are uncharged, polar, soluble, and do not interfere with cellular metabolism, even at high concentrations. These compatible osmolytes play a key role in maintaining the low intracellular osmotic potential of plants and in protecting cell structures. There is increasing evidence that proline accumulation in plants under saline conditions which is a primary defense response to maintain the osmotic pressure in cells, in particular high soil salinity and drought, which acts as an active osmolyte. Under non stress condition the levels of proline and free amino acids were low but as the salt concentration increased the concentration of proline and free amino acid increased. Sneha has found that proline and free amino acid contents increased with increase in salt concentration, and acted as compatible solutes to protect the cellular macromolecules and to maintain osmotic balance within the cell via continuous water influx (Sneha et al. 2013).

The synthesis and accumulation of compatible solute compounds is one of the strategies that plant cells use to increase tolerance of salinity stress. In the present study, through the KEGG analysis, we showed that free amino acid metabolism and carbohydrate metabolism were up-regulated (Table S6). Our results showed that kenaf is salt tolerant, and that free amino acid and sugar are important compounds for salt resistance in Zhonghongma variety 16.

### Transcription factors of salinity tolerance

Regulation of gene expression in salinity stress includes a wide array of mechanisms that are used by plants to up- or down-regulate the production of specific gene products (protein or RNA). Transcription factors are considered as the most important regulators that control gene expressions, including bZIP, WRKY, NAC, HD-Zip, and DREB. These transcription factors are capable of controlling the expression of a broad range of target genes by binding to the specific cis-acting element in the promoters of target genes. Johnson et al. (2002) found that the expression of bZIP genes was up-regulated in a salt-sensitive wheat cultivar under salinity stress. Overexpression of the NAC transcrption factor in both rice and wheat confers salt tolerance. Salt stress in Arabidopsis caused the up-regulation of the transcription factor AtWRKY8, which directly binds with the promoter of RD29A (Hu et al. 2013).

In the present study, we found transcrption factors NAC, BZIP AP2/EREBP, ARF, AP2/ERF, Bhlh, and TCP were up-regulated. But 8 NAC transcription factors and 2 WRKY transcription factors were found to be down-regulated. The results showed that these transcription factors play an important role in the salinity tolerance mechanisms of kenaf.

In conclusion, our study suggests that multiple pathways are involved in salinity tolerance in kenaf. The transcriptome sequencing and comparative transcriptome analysis will serve as a valuable resource to support genome analysis, develop expression arrays and will provide useful information for a better understanding of the physiological and molecular processes involved in salt tolerance in kenaf.

## Electronic supplementary material

Below is the link to the electronic supplementary material.

Supplementary material 1 (DOCX 14 KB)

Supplementary material 2 (DOCX 17 KB)

Supplementary material 3 (XLSX 20 KB)

Supplementary material 4 (DOCX 45 KB)

Supplementary material 5 (DOCX 21 KB)

Supplementary material 6 (DOCX 18 KB)

Supplementary material 7 (DOCX 18 KB)

Supplementary material 8 (DOCX 19 KB)
