# Supplementary material for: RNA-seq for comparative transcript profiling of kenaf under salinity stress
Source: J Plant Res. 2016 Dec 20;130(2):365–72. doi: 10.1007/s10265-016-0898-9 (PMC5318473; doi:10.1007/s10265-016-0898-9)
Supplement: Supplementary file 1 — Supplementary material 1 (DOCX 14 KB) [file 10265_2016_898_MOESM1_ESM.docx]

**Electric supplementary materials**

**Title:**

RNA-seq for comparative transcript profiling of kenaf under salinity stress

**Authors:**

Hui Li, Defang Li*, Anguo Chen, Huijuan Tang, Jianjun Li, Siqi Huang

**Journal:**

Journal of Plant Research

**Corresponding author:**

Defang Li

**Affiliation, Address, Country**

Institute of Bast Fiber Crops, Chinese Academy of Agricultural Sciences, Post Code 410205, No. 348 West Xianjiahu Road, Changsha, China

Tel: 0731-88998538

Fax : 0731-88998528

E-mail: [chinakenaf@126.com](mailto:chinakenaf@126.com)

**Content:**

**Tables. S1–S7**
