# Supplementary material for: RNA-seq for comparative transcript profiling of kenaf under salinity stress
Source: J Plant Res. 2016 Dec 20;130(2):365–72. doi: 10.1007/s10265-016-0898-9 (PMC5318473; doi:10.1007/s10265-016-0898-9)
Supplement: Supplementary file 2 — Supplementary material 2 (DOCX 17 KB) [file 10265_2016_898_MOESM2_ESM.docx]

**Table S1** Primers used the in the real-time PCR analysis

| Gene | Primer-F(5'-3') | Primer-R(5'-3') |
| --- | --- | --- |
| CL2927.Contig1 | TGGGTGCTTGAAGAAATC | TCCGCATGTAATCTGTAT |
| CL554.Contig12 | GCTATTTCTCACGGTTGC | CAGTGGACAGCACGTTTAT |
| Unigene27652 | CGGAGAAGGAGTGGTGAA | CACGAACGGTTGAATGTC |
| CL3883.Contig4 | GGTGCGGAGGAATTAGTA | ATGAAAGCAAATCGTGGT |
| Unigene13798 | AGAGTATGGTCCTTGTTG | CGTAATACAGTGGCTAAA |
| CL3599.Contig1 | ATAAAGAAGCAATGGGAACA | CACCGGAGATGGAGTTGA |
| CL541.Contig2 | AACCTTTCTGCCTTGAGT | AAATTGGCTGAGCTGTAC |
| CL9910.Contig3 | TGGCAGTGAAGTCGTGGTT | GAAGAGTTGGTGGTGGTAT |
| CL6853.Contig2 | AGGTGGAATGATGGTAATG | CTTGTCCGAAATCTGTCTC |
| CL897.Contig4 | TTCCCACCACTCCCTACT | CTCCGAGCACAAGCAAAT |
| Unigene10019 | TCTACTCGGAACAGGTCTC | GGTTATACATCTCGGCAAT |
| Unigene9404 | GATAACCGCATCAACAGG | AGCCCAAATCCAAGTAAA |
| Unigene7141 | ATTCATCAGTCAAGCCAAGA | TGCCTGCTACATCCAACT |
| CL184.Contig22 | ACAGTTCAAAGAAGGGAC | ATCGAAAGATTCACCAGA |
| Unigene28843 | CCCGACTCCGAGAAGAACC | ACGAGACGACCCGCTGAA |
| Unigene1983 | CAACTCCCAAATAACCCA | CAACGGCTATGATCCTAAA |
| Unigene17637 | TGACTATGGCTGGGACAC | ATCAGCACGACTTGGAAT |
| Unigene9851 | CAAGGATGGCAAGTAGAAG | AAAGTTAGTGGCGAAACG |
| CL5512.Contig1 | AGGAGTGTACCGAAGGAGA | AAGGTGGTGGCAACTTTAT |
| CL8228.Contig1 | TGTTTGAGGTGCCAGAAG | CAAGTAAAGCGAGGAAGTG |
| Actin | CAGGCAGTTCTTTCTTTGT | ATCCTCCAATCCAGACACT |
