# Supplementary material for: RNA-seq for comparative transcript profiling of kenaf under salinity stress
Source: J Plant Res. 2016 Dec 20;130(2):365–72. doi: 10.1007/s10265-016-0898-9 (PMC5318473; doi:10.1007/s10265-016-0898-9)
Supplement: Supplementary file 4 — Supplementary material 4 (DOCX 45 KB) [file 10265_2016_898_MOESM4_ESM.docx]

| **Table S3** Molecular-function category of up-regulated unigenes | |
| --- | --- |
| Gene Ontology term | Cluster frequency |
| oxidoreductase activity | 225 of 971 in the list |
| chlorophyll binding | 15 of 971 in the list |
| aminomethyltransferase activity | 8 of 971 in the list |
| pigment binding | 6 of 971 in the list |
| tetrapyrrole binding | 39 of 971 in the list |
| catalytic activity | 674 of 971 in the list |
| adenylyl-sulfate reductase (glutathione) activity | 6 of 971 in the list |
| inositol 3-alpha-galactosyltransferase activity | 7 of 971 in the list |
| transferase activity, transferring hexosyl groups | 57 of 971 in the list |
| xanthophyll binding | 5 of 971 in the list |
| water transmembrane transporter activity | 8 of 971 in the list |
| water channel activity | 8 of 971 in the list |
| glucosyltransferase activity | 33 of 971 in the list |
| adenylyl-sulfate reductase activity | 5 of 971 in the list |
| hydrolase activity, hydrolyzing O-glycosyl compounds | 42 of 971 in the list |
| trans-zeatin O-beta-D-glucosyltransferase activity | 9 of 971 in the list |
| cis-zeatin O-beta-D-glucosyltransferase activity | 9 of 971 in the list |
| quercetin 7-O-glucosyltransferase activity | 10 of 971 in the list |
| cofactor binding | 64 of 971 in the list |
| anthocyanidin 3-O-glucosyltransferase activity | 9 of 971 in the list |
| hydrolase activity, acting on glycosyl bonds | 44 of 971 in the list |
| UDP-glucosyltransferase activity | 29 of 971 in the list |
| oxidoreductase activity, acting on a sulfur group of donors, disulfide as acceptor | 11 of 971 in the list |
| glycine dehydrogenase (decarboxylating) activity | 5 of 971 in the list |
| oxidoreductase activity, acting on the CH-NH2 group of donors, disulfide as acceptor | 5 of 971 in the list |
| glyceraldehyde-3-phosphate dehydrogenase (NADP+) (phosphorylating) activity | 5 of 971 in the list |
| antioxidant activity | 20 of 971 in the list |
| quercetin 3-O-glucosyltransferase activity | 9 of 971 in the list |
| fructose-bisphosphate aldolase activity | 7 of 971 in the list |
| pyridoxal phosphate binding | 20 of 971 in the list |
| oxidoreductase activity, acting on the aldehyde or oxo group of donors | 21 of 971 in the list |
| quercetin 4'-O-glucosyltransferase activity | 9 of 971 in the list |
| poly-pyrimidine tract binding | 9 of 971 in the list |
| poly(U) RNA binding | 9 of 971 in the list |
| drug transmembrane transporter activity | 17 of 971 in the list |
| oxidoreductase activity, acting on the aldehyde or oxo group of donors, NAD or NADP as acceptor | 17 of 971 in the list |
| oxidoreductase activity, acting on CH-OH group of donors | 33 of 971 in the list |
| aldehyde-lyase activity | 8 of 971 in the list |
| NADH dehydrogenase (plastoquinone) activity | 3 of 971 in the list |
| UDP-glycosyltransferase activity | 37 of 971 in the list |
| drug transporter activity | 17 of 971 in the list |
| lyase activity | 47 of 971 in the list |
| transaminase activity | 13 of 971 in the list |
| transferase activity, transferring nitrogenous groups | 13 of 971 in the list |
| NADPH dehydrogenase activity | 4 of 971 in the list |
| peroxidase activity | 14 of 971 in the list |
| oxidoreductase activity, acting on peroxide as acceptor | 14 of 971 in the list |
| single-stranded RNA binding | 12 of 971 in the list |
| NADP binding | 14 of 971 in the list |
| carbon-sulfur lyase activity | 11 of 971 in the list |
| monooxygenase activity | 30 of 971 in the list |
| glycine:2-oxoglutarate aminotransferase activity | 3 of 971 in the list |
| glutamate-ammonia ligase activity | 4 of 971 in the list |
| oxidoreductase activity, acting on paired donors, with incorporation or reduction of molecular oxygen | 39 of 971 in the list |
| gamma-glutamyltransferase activity | 3 of 971 in the list |
| urea transmembrane transporter activity | 3 of 971 in the list |
| secondary active transmembrane transporter activity | 32 of 971 in the list |
| superoxide dismutase activity | 6 of 971 in the list |
| oxidoreductase activity, acting on superoxide radicals as acceptor | 6 of 971 in the list |
| oxidoreductase activity, acting on the CH-OH group of donors, NAD or NADP as acceptor | 28 of 971 in the list |
| L-alanine:2-oxoglutarate aminotransferase activity | 4 of 971 in the list |
| electron transporter, transferring electrons within the cyclic electron transport pathway of photosynthesis activity | 4 of 971 in the list |
| alanine-oxo-acid transaminase activity | 4 of 971 in the list |
| calcium:hydrogen antiporter activity | 5 of 971 in the list |
| ammonia ligase activity | 4 of 971 in the list |
| acid-ammonia (or amide) ligase activity | 4 of 971 in the list |
| transferase activity, transferring glycosyl groups | 60 of 971 in the list |
| indole-3-acetic acid amido synthetase activity | 5 of 971 in the list |
| auxin transmembrane transporter activity | 8 of 971 in the list |
| isoprenoid binding | 5 of 971 in the list |
| metal ion:hydrogen antiporter activity | 5 of 971 in the list |
| phosphate ion transmembrane-transporting ATPase activity | 4 of 971 in the list |
| calcium:cation antiporter activity | 5 of 971 in the list |
| omega-3 fatty acid desaturase activity | 3 of 971 in the list |
| phosphoadenylyl-sulfate reductase (thioredoxin) activity | 2 of 971 in the list |
| plastoquinol--plastocyanin reductase activity | 2 of 971 in the list |
| oxidoreductase activity, acting on diphenols and related substances as donors, with copper protein as acceptor | 2 of 971 in the list |
| coenzyme binding | 41 of 971 in the list |
| phosphate ion transmembrane transporter activity | 5 of 971 in the list |
| 1-aminocyclopropane-1-carboxylate synthase activity | 5 of 971 in the list |
| cellulose synthase (UDP-forming) activity | 8 of 971 in the list |
| fructose 1,6-bisphosphate 1-phosphatase activity | 4 of 971 in the list |
| copper ion binding | 28 of 971 in the list |
| electron carrier activity | 32 of 971 in the list |
| ferric-chelate reductase activity | 3 of 971 in the list |
| oxidoreductase activity, oxidizing metal ions, NAD or NADP as acceptor | 3 of 971 in the list |
| cellulose synthase activity | 8 of 971 in the list |
| galactosyltransferase activity | 9 of 971 in the list |
| antiporter activity | 19 of 971 in the list |
| UDP-galactosyltransferase activity | 7 of 971 in the list |
| alanine-glyoxylate transaminase activity | 3 of 971 in the list |
| phosphomethylethanolamine N-methyltransferase activity | 3 of 971 in the list |
| dihydroorotase activity | 2 of 971 in the list |
| proline dehydrogenase activity | 2 of 971 in the list |
| beta-phosphoglucomutase activity | 2 of 971 in the list |
| L-galactose dehydrogenase activity | 2 of 971 in the list |
| ribulose-bisphosphate carboxylase activity | 2 of 971 in the list |
| pyridoxal 4-dehydrogenase activity | 2 of 971 in the list |
| aldo-keto reductase (NADP) activity | 6 of 971 in the list |
| disaccharide transmembrane transporter activity | 5 of 971 in the list |
| transmembrane transporter activity | 88 of 971 in the list |
| amylase activity | 7 of 971 in the list |
| glutathione gamma-glutamylcysteinyltransferase activity | 3 of 971 in the list |
| oligosaccharide transmembrane transporter activity | 5 of 971 in the list |
| NAD binding | 12 of 971 in the list |
| carboxylesterase activity | 9 of 971 in the list |
| transporter activity | 109 of 971 in the list |
| oxygen evolving activity | 2 of 971 in the list |
| electron transporter, transferring electrons within the noncyclic electron transport pathway of photosynthesis activity | 2 of 971 in the list |
| electron transporter, transferring electrons within cytochrome b6/f complex of photosystem II activity | 2 of 971 in the list |
| mitochondrial light strand promoter anti-sense binding | 2 of 971 in the list |
| carbohydrate transmembrane transporter activity | 13 of 971 in the list |
| carbohydrate transporter activity | 13 of 971 in the list |
| sugar-phosphatase activity | 4 of 971 in the list |
| carbonate dehydratase activity | 5 of 971 in the list |
| cinnamyl-alcohol dehydrogenase activity | 3 of 971 in the list |
| transferase activity, transferring acyl groups, acyl groups converted into alkyl on transfer | 5 of 971 in the list |
| heme binding | 22 of 971 in the list |
| O-methyltransferase activity | 6 of 971 in the list |
| glycolate oxidase activity | 3 of 971 in the list |
| auxin influx transmembrane transporter activity | 3 of 971 in the list |
| oxidoreductase activity, acting on a sulfur group of donors | 15 of 971 in the list |
| oxidoreductase activity, acting on diphenols and related substances as donors | 7 of 971 in the list |
| intramolecular oxidoreductase activity, interconverting aldoses and ketoses | 5 of 971 in the list |
| oxygen binding | 8 of 971 in the list |
| 1,4-alpha-glucan branching enzyme activity | 2 of 971 in the list |
| formamidase activity | 2 of 971 in the list |
| oxidoreductase activity, acting on the CH-CH group of donors | 42 of 971 in the list |
| cation binding | 216 of 971 in the list |
| intramolecular oxidoreductase activity | 8 of 971 in the list |
| substrate-specific transmembrane transporter activity | 73 of 971 in the list |
| beta-galactosidase activity | 5 of 971 in the list |
| unfolded protein binding | 15 of 971 in the list |
| sugar transmembrane transporter activity | 12 of 971 in the list |
| (S)-2-hydroxy-acid oxidase activity | 3 of 971 in the list |
| alcohol dehydrogenase (NADP+) activity | 3 of 971 in the list |
| phenylalanine ammonia-lyase activity | 3 of 971 in the list |
| iron ion binding | 27 of 971 in the list |
| active transmembrane transporter activity | 50 of 971 in the list |
| manganese ion binding | 7 of 971 in the list |
| dioxygenase activity | 17 of 971 in the list |
| chitinase activity | 4 of 971 in the list |
| 2-isopropylmalate synthase activity | 2 of 971 in the list |
| phosphoglycerate kinase activity | 2 of 971 in the list |
| dipeptidase activity | 2 of 971 in the list |
| aryl-alcohol dehydrogenase (NAD+) activity | 2 of 971 in the list |
| oxidoreductase activity, acting on diphenols and related substances as donors, oxygen as acceptor | 5 of 971 in the list |
| inorganic anion transmembrane transporter activity | 12 of 971 in the list |
| oxidoreductase activity, acting on the CH-NH2 group of donors | 7 of 971 in the list |
| isomerase activity | 29 of 971 in the list |
| xyloglucan:xyloglucosyl transferase activity | 5 of 971 in the list |
| alcohol dehydrogenase (NAD) activity | 3 of 971 in the list |
| dihydrokaempferol 4-reductase activity | 4 of 971 in the list |
| oxidoreductase activity, acting on the CH-CH group of donors, NAD or NADP as acceptor | 37 of 971 in the list |
| ferredoxin-NADP+ reductase activity | 2 of 971 in the list |
| hydroxymethylglutaryl-CoA synthase activity | 2 of 971 in the list |
| maltose transmembrane transporter activity | 2 of 971 in the list |
| alternative oxidase activity | 2 of 971 in the list |
| (+)-abscisic acid 8'-hydroxylase activity | 2 of 971 in the list |
| glycerol transmembrane transporter activity | 2 of 971 in the list |
| peptide-methionine (R)-S-oxide reductase activity | 2 of 971 in the list |
| caffeate O-methyltransferase activity | 2 of 971 in the list |
| oxidoreductase activity, acting on paired donors, with incorporation or reduction of molecular oxygen, NAD(P)H as one donor, and incorporation of one atom of oxygen | 11 of 971 in the list |
| phosphoethanolamine N-methyltransferase activity | 3 of 971 in the list |
| ammonia-lyase activity | 3 of 971 in the list |
| methyl salicylate esterase activity | 3 of 971 in the list |
| xenobiotic-transporting ATPase activity | 6 of 971 in the list |
| xenobiotic transporter activity | 6 of 971 in the list |
| oxidoreductase activity, acting on the CH-NH group of donors | 5 of 971 in the list |
| oxidoreductase activity, oxidizing metal ions | 3 of 971 in the list |
| phosphorelay sensor kinase activity | 8 of 971 in the list |
| amide transmembrane transporter activity | 4 of 971 in the list |
| transferase activity, transferring acyl groups | 33 of 971 in the list |
| thiosulfate sulfurtransferase activity | 2 of 971 in the list |
| carotenoid dioxygenase activity | 2 of 971 in the list |
| isoamylase activity | 2 of 971 in the list |
| hexitol dehydrogenase activity | 2 of 971 in the list |
| 9-cis-epoxycarotenoid dioxygenase activity | 2 of 971 in the list |
| mannitol dehydrogenase activity | 2 of 971 in the list |
| macrolide binding | 4 of 971 in the list |
| FK506 binding | 4 of 971 in the list |
| alpha-amylase activity | 3 of 971 in the list |
| glutathione peroxidase activity | 3 of 971 in the list |
| methyl jasmonate esterase activity | 3 of 971 in the list |
| transferase activity, transferring sulfur-containing groups | 5 of 971 in the list |
| phospholipase C activity | 3 of 971 in the list |
| lactoylglutathione lyase activity | 4 of 971 in the list |
| triose-phosphate isomerase activity | 2 of 971 in the list |
| glycogen phosphorylase activity | 2 of 971 in the list |
| photoreceptor activity | 6 of 971 in the list |
| carbohydrate binding | 14 of 971 in the list |
| acid phosphatase activity | 5 of 971 in the list |
| 2-alkenal reductase [NAD(P)] activity | 32 of 971 in the list |
| sulfate adenylyltransferase (ATP) activity | 3 of 971 in the list |
| secondary active sulfate transmembrane transporter activity | 3 of 971 in the list |
| methyl indole-3-acetate esterase activity | 3 of 971 in the list |
| metal ion binding | 204 of 971 in the list |
| glycogen debranching enzyme activity | 2 of 971 in the list |
| phosphoglucomutase activity | 2 of 971 in the list |
| peroxiredoxin activity | 2 of 971 in the list |
| substrate-specific channel activity | 11 of 971 in the list |
| core RNA polymerase binding transcription factor activity | 3 of 971 in the list |
| core DNA-dependent RNA polymerase binding promoter specificity activity | 3 of 971 in the list |
| plastid sigma factor activity | 3 of 971 in the list |
| sulfate adenylyltransferase activity | 3 of 971 in the list |
| transferase activity, transferring amino-acyl groups | 3 of 971 in the list |
| sigma factor activity | 3 of 971 in the list |
| channel activity | 11 of 971 in the list |
| passive transmembrane transporter activity | 11 of 971 in the list |
| methyltransferase activity | 28 of 971 in the list |
| oxidoreductase activity, acting on NAD(P)H | 13 of 971 in the list |
| core promoter proximal region sequence-specific DNA binding | 2 of 971 in the list |
| core promoter proximal region DNA binding | 2 of 971 in the list |
| delta12-fatty acid dehydrogenase activity | 2 of 971 in the list |
| oxidoreductase activity, acting on iron-sulfur proteins as donors, NAD or NADP as acceptor | 2 of 971 in the list |
| substrate-specific transporter activity | 78 of 971 in the list |
| protein histidine kinase activity | 8 of 971 in the list |
| phosphotransferase activity, nitrogenous group as acceptor | 8 of 971 in the list |
| galactosidase activity | 5 of 971 in the list |
| carbon-carbon lyase activity | 14 of 971 in the list |
| sucrose transmembrane transporter activity | 3 of 971 in the list |
| starch synthase activity | 3 of 971 in the list |
| protochlorophyllide reductase activity | 3 of 971 in the list |
| sulfurtransferase activity | 3 of 971 in the list |
| transferase activity, transferring one-carbon groups | 28 of 971 in the list |
| glucosidase activity | 7 of 971 in the list |
| protein dimerization activity | 38 of 971 in the list |
| beta-amylase activity | 4 of 971 in the list |
| oxidoreductase activity, acting on the CH-NH group of donors, NAD or NADP as acceptor | 3 of 971 in the list |
| alditol:NADP+ 1-oxidoreductase activity | 2 of 971 in the list |
| drug binding | 4 of 971 in the list |
| intramolecular transferase activity, phosphotransferases | 4 of 971 in the list |
| sphingosine hydroxylase activity | 2 of 971 in the list |
| alpha-L-fucosidase activity | 2 of 971 in the list |
| galactose transmembrane transporter activity | 2 of 971 in the list |
| fucosidase activity | 2 of 971 in the list |
| sucrose synthase activity | 2 of 971 in the list |
| symporter activity | 10 of 971 in the list |
| intramolecular transferase activity | 9 of 971 in the list |
| 3-chloroallyl aldehyde dehydrogenase activity | 4 of 971 in the list |
| aldehyde dehydrogenase (NAD) activity | 4 of 971 in the list |
| arsenate reductase (glutaredoxin) activity | 2 of 971 in the list |
| phosphoenolpyruvate carboxylase activity | 2 of 971 in the list |
| oxidoreductase activity, acting on phosphorus or arsenic in donors | 2 of 971 in the list |
| oxidoreductase activity, acting on phosphorus or arsenic in donors, disulfide as acceptor | 2 of 971 in the list |
| calcium ion transmembrane transporter activity | 6 of 971 in the list |
| neutral amino acid transmembrane transporter activity | 3 of 971 in the list |
| oxidoreductase activity, acting on paired donors, with incorporation or reduction of molecular oxygen, reduced flavin or flavoprotein as one donor, and incorporation of one atom of oxygen | 3 of 971 in the list |
| nutrient reservoir activity | 3 of 971 in the list |
| aromatase activity | 3 of 971 in the list |
| carboxylic ester hydrolase activity | 16 of 971 in the list |
| ATP-dependent peptidase activity | 5 of 971 in the list |
| NAD+ diphosphatase activity | 2 of 971 in the list |
| 3-beta-hydroxy-delta5-steroid dehydrogenase activity | 2 of 971 in the list |
| diacylglycerol O-acyltransferase activity | 2 of 971 in the list |
| ribose-5-phosphate isomerase activity | 2 of 971 in the list |
| carbohydrate phosphatase activity | 5 of 971 in the list |
| proton-transporting ATPase activity, rotational mechanism | 5 of 971 in the list |
| glyceraldehyde-3-phosphate dehydrogenase (NAD+) (phosphorylating) activity | 2 of 971 in the list |
| glycylpeptide N-tetradecanoyltransferase activity | 2 of 971 in the list |
| cinnamoyl-CoA reductase activity | 2 of 971 in the list |
| intramolecular oxidoreductase activity, transposing C=C bonds | 2 of 971 in the list |
| myristoyltransferase activity | 2 of 971 in the list |
| oxidoreductase activity, acting on NAD(P)H, quinone or similar compound as acceptor | 7 of 971 in the list |
| alpha-glucosidase activity | 3 of 971 in the list |
| calcium-dependent phospholipid binding | 3 of 971 in the list |
| oxidoreductase activity, acting on the CH-OH group of donors, oxygen as acceptor | 3 of 971 in the list |
| flavin adenine dinucleotide binding | 11 of 971 in the list |
| pyruvate dehydrogenase activity | 3 of 971 in the list |
| pyruvate dehydrogenase (acetyl-transferring) activity | 3 of 971 in the list |
| NADH dehydrogenase (quinone) activity | 4 of 971 in the list |
| phosphatidylcholine 1-acylhydrolase activity | 2 of 971 in the list |
| auxin:hydrogen symporter activity | 2 of 971 in the list |
| arsenate reductase activity | 2 of 971 in the list |
| oxidoreductase activity, acting on single donors with incorporation of molecular oxygen, incorporation of two atoms of oxygen | 7 of 971 in the list |
| oxidoreductase activity, acting on single donors with incorporation of molecular oxygen | 9 of 971 in the list |
| auxin efflux transmembrane transporter activity | 3 of 971 in the list |
| sulfate transmembrane transporter activity | 3 of 971 in the list |
| efflux transmembrane transporter activity | 4 of 971 in the list |
| oxidoreductase activity, acting on the CH-CH group of donors, oxygen as acceptor | 4 of 971 in the list |
| oxidoreductase activity, acting on the aldehyde or oxo group of donors, oxygen as acceptor | 2 of 971 in the list |
| acylglycerol O-acyltransferase activity | 3 of 971 in the list |
| phosphotransferase activity, carboxyl group as acceptor | 3 of 971 in the list |
| C-4 methylsterol oxidase activity | 2 of 971 in the list |
| FMN binding | 7 of 971 in the list |
| acidic amino acid transmembrane transporter activity | 3 of 971 in the list |
| sulfur compound transmembrane transporter activity | 3 of 971 in the list |
| transcription regulatory region DNA binding | 5 of 971 in the list |
| regulatory region DNA binding | 5 of 971 in the list |
| regulatory region nucleic acid binding | 5 of 971 in the list |
| solute:hydrogen antiporter activity | 5 of 971 in the list |
| NAD(P)H oxidase activity | 2 of 971 in the list |
| secologanin synthase activity | 2 of 971 in the list |
| oxidoreductase activity, acting on NAD(P)H, oxygen as acceptor | 2 of 971 in the list |
| lipase activity | 10 of 971 in the list |
| hydro-lyase activity | 10 of 971 in the list |
| carbon-oxygen lyase activity | 13 of 971 in the list |
| oxidoreductase activity, acting on paired donors, with oxidation of a pair of donors resulting in the reduction of molecular oxygen to two molecules of water | 3 of 971 in the list |
| blue light photoreceptor activity | 4 of 971 in the list |
| hexose transmembrane transporter activity | 2 of 971 in the list |
| hydrolase activity, acting on carbon-nitrogen (but not peptide) bonds, in cyclic amides | 2 of 971 in the list |
| borate transmembrane transporter activity | 2 of 971 in the list |
| serine-type carboxypeptidase activity | 3 of 971 in the list |
| auxin binding | 3 of 971 in the list |
| lipid binding | 18 of 971 in the list |
| phospholipase activity | 6 of 971 in the list |
| 3-oxoacyl-[acyl-carrier-protein] synthase activity | 2 of 971 in the list |
| glucose-1-phosphate adenylyltransferase activity | 2 of 971 in the list |
| oxidoreductase activity, acting on iron-sulfur proteins as donors | 2 of 971 in the list |
| glutathione transferase activity | 3 of 971 in the list |
| steroid dehydrogenase activity | 3 of 971 in the list |
| oxidoreductase activity, acting on the aldehyde or oxo group of donors, disulfide as acceptor | 3 of 971 in the list |
| microtubule-severing ATPase activity | 7 of 971 in the list |
| carbon-nitrogen lyase activity | 3 of 971 in the list |
| ATPase activity, coupled to transmembrane movement of ions, rotational mechanism | 5 of 971 in the list |
| identical protein binding | 20 of 971 in the list |
| phosphoenolpyruvate carboxykinase activity | 2 of 971 in the list |
| galactolipase activity | 2 of 971 in the list |
| retinyl-palmitate esterase activity | 2 of 971 in the list |
| cation:cation antiporter activity | 5 of 971 in the list |
| oxidoreductase activity, acting on paired donors, with incorporation or reduction of molecular oxygen, 2-oxoglutarate as one donor, and incorporation of one atom each of oxygen into both donors | 10 of 971 in the list |
| carboxypeptidase activity | 4 of 971 in the list |
| protein histidine kinase binding | 4 of 971 in the list |
| monosaccharide transmembrane transporter activity | 2 of 971 in the list |
| ER retention sequence binding | 2 of 971 in the list |
| transcription regulatory region sequence-specific DNA binding | 3 of 971 in the list |
| hydrogen ion transmembrane transporter activity | 13 of 971 in the list |
| cis-trans isomerase activity | 7 of 971 in the list |
| sucrose alpha-glucosidase activity | 2 of 971 in the list |
| pyruvate kinase activity | 2 of 971 in the list |
| 1-phosphatidylinositol binding | 2 of 971 in the list |
| potassium ion binding | 2 of 971 in the list |
| alkali metal ion binding | 2 of 971 in the list |
| O-acyltransferase activity | 6 of 971 in the list |
| NADH dehydrogenase activity | 4 of 971 in the list |
| lipoxygenase activity | 2 of 971 in the list |
| beta-glucosidase activity | 4 of 971 in the list |
| NADPH:quinone reductase activity | 2 of 971 in the list |
| polyol transmembrane transporter activity | 2 of 971 in the list |
| pattern binding | 3 of 971 in the list |
| polysaccharide binding | 3 of 971 in the list |
| adenylyltransferase activity | 4 of 971 in the list |
| solute:cation antiporter activity | 5 of 971 in the list |
| peptidyl-prolyl cis-trans isomerase activity | 6 of 971 in the list |
| transferase activity, transferring acyl groups other than amino-acyl groups | 22 of 971 in the list |
| nucleotide diphosphatase activity | 2 of 971 in the list |
| sulfotransferase activity | 2 of 971 in the list |
| peptide binding | 3 of 971 in the list |
| divalent inorganic cation transmembrane transporter activity | 8 of 971 in the list |
| oxidoreductase activity, acting on single donors with incorporation of molecular oxygen, incorporation of one atom of oxygen (internal monooxygenases or internal mixed function oxidases) | 3 of 971 in the list |
| beta-fructofuranosidase activity | 2 of 971 in the list |
| phosphorylase activity | 2 of 971 in the list |
| ubiquinol-cytochrome-c reductase activity | 2 of 971 in the list |
| alcohol transmembrane transporter activity | 2 of 971 in the list |
| 4-coumarate-CoA ligase activity | 2 of 971 in the list |
| oxidoreductase activity, acting on diphenols and related substances as donors, cytochrome as acceptor | 2 of 971 in the list |
| organic hydroxy compound transmembrane transporter activity | 2 of 971 in the list |
| serine-type peptidase activity | 11 of 971 in the list |
| serine hydrolase activity | 11 of 971 in the list |
| CoA-ligase activity | 4 of 971 in the list |
| protein homodimerization activity | 11 of 971 in the list |
| protein disulfide oxidoreductase activity | 6 of 971 in the list |
| cellulase activity | 2 of 971 in the list |
| calcium ion binding | 21 of 971 in the list |
| transition metal ion binding | 123 of 971 in the list |
| terpene synthase activity | 2 of 971 in the list |
| malate dehydrogenase activity | 2 of 971 in the list |
| steroid dehydrogenase activity, acting on the CH-OH group of donors, NAD or NADP as acceptor | 2 of 971 in the list |
| luciferin monooxygenase activity | 2 of 971 in the list |
| Photinus-luciferin 4-monooxygenase (ATP-hydrolyzing) activity | 2 of 971 in the list |
| serine-type exopeptidase activity | 3 of 971 in the list |
| aspartic-type endopeptidase activity | 6 of 971 in the list |
| L-ascorbate oxidase activity | 2 of 971 in the list |
| UDP-N-acetylmuramate dehydrogenase activity | 2 of 971 in the list |
| purine nucleoside transmembrane transporter activity | 2 of 971 in the list |
| aspartic-type peptidase activity | 6 of 971 in the list |
| hormone binding | 3 of 971 in the list |
| solute:cation symporter activity | 7 of 971 in the list |
| acid-thiol ligase activity | 4 of 971 in the list |
| anion transmembrane transporter activity | 20 of 971 in the list |
| monovalent inorganic cation transmembrane transporter activity | 17 of 971 in the list |
| quinone binding | 2 of 971 in the list |
| 2 iron, 2 sulfur cluster binding | 3 of 971 in the list |
| translation elongation factor activity | 4 of 971 in the list |
| aldehyde dehydrogenase [NAD(P)+] activity | 2 of 971 in the list |
| pseudouridine synthase activity | 2 of 971 in the list |
| fatty acid synthase activity | 4 of 971 in the list |
| transferase activity, transferring alkyl or aryl (other than methyl) groups | 7 of 971 in the list |
| pectinesterase activity | 3 of 971 in the list |
| signal sequence binding | 2 of 971 in the list |
| carboxylic acid transmembrane transporter activity | 7 of 971 in the list |
| protein kinase C activity | 3 of 971 in the list |
| polygalacturonase activity | 2 of 971 in the list |
| oxidosqualene cyclase activity | 2 of 971 in the list |
| amide binding | 3 of 971 in the list |
| disulfide oxidoreductase activity | 6 of 971 in the list |
| organic acid transmembrane transporter activity | 7 of 971 in the list |
| ion binding | 351 of 971 in the list |
| nucleoside transmembrane transporter activity | 2 of 971 in the list |
| carbon-oxygen lyase activity, acting on phosphates | 2 of 971 in the list |
| microtubule binding | 3 of 971 in the list |
| exopeptidase activity | 7 of 971 in the list |
| ADP binding | 2 of 971 in the list |
| single-stranded DNA binding | 2 of 971 in the list |
| proton-transporting ATP synthase activity, rotational mechanism | 2 of 971 in the list |
| ligase activity, forming carbon-sulfur bonds | 4 of 971 in the list |
| amino acid transmembrane transporter activity | 5 of 971 in the list |
| protein kinase binding | 5 of 971 in the list |
| anion transmembrane-transporting ATPase activity | 4 of 971 in the list |
| aspartyl esterase activity | 2 of 971 in the list |
| carboxy-lyase activity | 5 of 971 in the list |
| metalloendopeptidase activity | 5 of 971 in the list |
| actin filament binding | 3 of 971 in the list |
| N-methyltransferase activity | 4 of 971 in the list |
| C-acyltransferase activity | 2 of 971 in the list |
| oxidoreductase activity, acting on the CH-NH2 group of donors, oxygen as acceptor | 2 of 971 in the list |
| tubulin binding | 3 of 971 in the list |
| Ran GTPase binding | 2 of 971 in the list |
| magnesium ion transmembrane transporter activity | 2 of 971 in the list |
| ATPase activity, coupled to transmembrane movement of substances | 16 of 971 in the list |
| ATPase activity, coupled to movement of substances | 16 of 971 in the list |
| cation channel activity | 3 of 971 in the list |
| hydrolase activity, acting on acid anhydrides, catalyzing transmembrane movement of substances | 16 of 971 in the list |
| ATPase activity, coupled | 31 of 971 in the list |
| structural constituent of cytoskeleton | 3 of 971 in the list |
| kinase binding | 5 of 971 in the list |
| ion transmembrane transporter activity | 45 of 971 in the list |
| peptide transporter activity | 2 of 971 in the list |
| sugar:hydrogen symporter activity | 4 of 971 in the list |
| cation:sugar symporter activity | 4 of 971 in the list |
| MAP kinase activity | 3 of 971 in the list |
| solute:hydrogen symporter activity | 4 of 971 in the list |
| voltage-gated potassium channel activity | 2 of 971 in the list |
| signaling receptor activity | 9 of 971 in the list |
| inorganic cation transmembrane transporter activity | 21 of 971 in the list |
| voltage-gated cation channel activity | 2 of 971 in the list |
| damaged DNA binding | 2 of 971 in the list |
| endopeptidase activity | 17 of 971 in the list |
| protein binding transcription factor activity | 4 of 971 in the list |
| microtubule motor activity | 3 of 971 in the list |
| potassium channel activity | 2 of 971 in the list |
| enzyme inhibitor activity | 3 of 971 in the list |
| heat shock protein binding | 5 of 971 in the list |
| hydrolase activity | 194 of 971 in the list |
| GTPase activity | 9 of 971 in the list |
| iron-sulfur cluster binding | 6 of 971 in the list |
| metal cluster binding | 6 of 971 in the list |
| Ras GTPase binding | 2 of 971 in the list |
| small GTPase binding | 2 of 971 in the list |
| ATPase activity, coupled to transmembrane movement of ions | 10 of 971 in the list |
| triglyceride lipase activity | 2 of 971 in the list |
| protein tyrosine/serine/threonine phosphatase activity | 2 of 971 in the list |
| rRNA binding | 3 of 971 in the list |
| metallopeptidase activity | 6 of 971 in the list |
| ATPase activity | 34 of 971 in the list |
| nucleoside-triphosphatase activity | 53 of 971 in the list |
| carbohydrate derivative transporter activity | 3 of 971 in the list |
| serine-type endopeptidase activity | 5 of 971 in the list |
| magnesium ion binding | 6 of 971 in the list |
| structure-specific DNA binding | 3 of 971 in the list |
| S-adenosylmethionine-dependent methyltransferase activity | 6 of 971 in the list |
| metal ion transmembrane transporter activity | 13 of 971 in the list |
| phosphoric diester hydrolase activity | 3 of 971 in the list |
| hydrolase activity, acting on carbon-nitrogen (but not peptide) bonds, in linear amides | 3 of 971 in the list |
| P-P-bond-hydrolysis-driven transmembrane transporter activity | 17 of 971 in the list |
| guanyl-nucleotide exchange factor activity | 2 of 971 in the list |
| pyrophosphatase activity | 56 of 971 in the list |
| enzyme binding | 9 of 971 in the list |
| primary active transmembrane transporter activity | 17 of 971 in the list |
| receptor activity | 9 of 971 in the list |
| phosphorelay response regulator activity | 4 of 971 in the list |
| hydrolase activity, acting on acid anhydrides, in phosphorus-containing anhydrides | 56 of 971 in the list |
| cation transmembrane transporter activity | 28 of 971 in the list |
| hydrolase activity, acting on carbon-nitrogen (but not peptide) bonds | 5 of 971 in the list |
| racemase and epimerase activity | 2 of 971 in the list |
| clathrin binding | 2 of 971 in the list |
| hydrolase activity, acting on acid anhydrides | 56 of 971 in the list |
| GTPase binding | 2 of 971 in the list |
| nucleobase-containing compound transmembrane transporter activity | 3 of 971 in the list |
| DNA-directed RNA polymerase activity | 2 of 971 in the list |
| phospholipid binding | 8 of 971 in the list |
| voltage-gated ion channel activity | 2 of 971 in the list |
| voltage-gated channel activity | 2 of 971 in the list |
| peptidase activity, acting on L-amino acid peptides | 26 of 971 in the list |
| cation-transporting ATPase activity | 6 of 971 in the list |
| RNA polymerase activity | 2 of 971 in the list |
| motor activity | 3 of 971 in the list |
| organic anion transmembrane transporter activity | 8 of 971 in the list |
| nucleobase-containing compound kinase activity | 2 of 971 in the list |
| lipid transporter activity | 2 of 971 in the list |
| phosphate transmembrane transporter activity | 2 of 971 in the list |
| potassium ion transmembrane transporter activity | 3 of 971 in the list |
| peptidase activity | 29 of 971 in the list |
| N-acyltransferase activity | 4 of 971 in the list |
| cobalt ion binding | 2 of 971 in the list |
| ion channel activity | 3 of 971 in the list |
| actin binding | 4 of 971 in the list |
| transmembrane signaling receptor activity | 3 of 971 in the list |
| amino acid binding | 2 of 971 in the list |
| acetyltransferase activity | 4 of 971 in the list |
| gated channel activity | 2 of 971 in the list |
| ion gated channel activity | 2 of 971 in the list |
| ATPase activity, coupled to transmembrane movement of ions, phosphorylative mechanism | 2 of 971 in the list |
| receptor signaling protein serine/threonine kinase activity | 7 of 971 in the list |
| receptor signaling protein activity | 7 of 971 in the list |
| cytoskeletal protein binding | 6 of 971 in the list |
| transferase activity | 222 of 971 in the list |
| helicase activity | 8 of 971 in the list |
| aminoacyl-tRNA ligase activity | 2 of 971 in the list |
| ligase activity, forming carbon-oxygen bonds | 2 of 971 in the list |
| ligase activity, forming aminoacyl-tRNA and related compounds | 2 of 971 in the list |
| protein heterodimerization activity | 4 of 971 in the list |
| exonuclease activity | 2 of 971 in the list |
| thiolester hydrolase activity | 3 of 971 in the list |
| hydrolase activity, acting on ester bonds | 53 of 971 in the list |
| phosphatase activity | 22 of 971 in the list |
| calmodulin binding | 5 of 971 in the list |
| MAP kinase kinase kinase activity | 4 of 971 in the list |
| non-membrane spanning protein tyrosine kinase activity | 4 of 971 in the list |
| zinc ion binding | 68 of 971 in the list |
| ATP-dependent helicase activity | 4 of 971 in the list |
| purine NTP-dependent helicase activity | 4 of 971 in the list |
| signal transducer activity | 20 of 971 in the list |
| molecular transducer activity | 20 of 971 in the list |
| histone binding | 2 of 971 in the list |
| phosphatidylinositol binding | 2 of 971 in the list |
| phosphoric ester hydrolase activity | 25 of 971 in the list |
| ligase activity, forming carbon-nitrogen bonds | 20 of 971 in the list |
| N-acetyltransferase activity | 2 of 971 in the list |
| carboxylic acid binding | 2 of 971 in the list |
| nucleotidyltransferase activity | 7 of 971 in the list |
| chromatin binding | 9 of 971 in the list |
| small GTPase regulator activity | 2 of 971 in the list |
| GTP binding | 11 of 971 in the list |
| guanyl ribonucleotide binding | 11 of 971 in the list |
| guanyl nucleotide binding | 11 of 971 in the list |
| anion binding | 158 of 971 in the list |
| translation factor activity, nucleic acid binding | 5 of 971 in the list |
| GTPase regulator activity | 2 of 971 in the list |
| protein transporter activity | 3 of 971 in the list |
| nucleoside-triphosphatase regulator activity | 2 of 971 in the list |
| ATP binding | 113 of 971 in the list |
| adenyl ribonucleotide binding | 114 of 971 in the list |
| sequence-specific DNA binding transcription factor activity | 32 of 971 in the list |
| adenyl nucleotide binding | 114 of 971 in the list |
| structural molecule activity | 19 of 971 in the list |
| nucleic acid binding transcription factor activity | 32 of 971 in the list |
| protein tyrosine kinase activity | 11 of 971 in the list |
| enzyme regulator activity | 8 of 971 in the list |
| nucleotide binding | 202 of 971 in the list |
| nucleoside phosphate binding | 202 of 971 in the list |
| protein serine/threonine phosphatase activity | 4 of 971 in the list |
| acid-amino acid ligase activity | 13 of 971 in the list |
| small molecule binding | 202 of 971 in the list |
| protein binding | 138 of 971 in the list |
| ligase activity | 26 of 971 in the list |
| purine ribonucleoside triphosphate binding | 124 of 971 in the list |
| nuclease activity | 2 of 971 in the list |
| RNA binding | 32 of 971 in the list |
| phosphoprotein phosphatase activity | 8 of 971 in the list |
| ribonucleotide binding | 128 of 971 in the list |
| purine nucleoside binding | 125 of 971 in the list |
| purine ribonucleoside binding | 125 of 971 in the list |
| purine ribonucleotide binding | 125 of 971 in the list |
| ribonucleoside binding | 125 of 971 in the list |
| purine nucleotide binding | 125 of 971 in the list |
| nucleoside binding | 125 of 971 in the list |
| sequence-specific DNA binding | 9 of 971 in the list |
| ubiquitin-protein ligase activity | 8 of 971 in the list |
| small conjugating protein ligase activity | 8 of 971 in the list |
| structural constituent of ribosome | 9 of 971 in the list |
| protein serine/threonine kinase activity | 34 of 971 in the list |
| binding | 566 of 971 in the list |
| protein kinase activity | 48 of 971 in the list |
| heterocyclic compound binding | 313 of 971 in the list |
| organic cyclic compound binding | 313 of 971 in the list |
| DNA binding | 48 of 971 in the list |
| phosphotransferase activity, alcohol group as acceptor | 51 of 971 in the list |
| kinase activity | 65 of 971 in the list |
| nucleic acid binding | 92 of 971 in the list |
| transferase activity, transferring phosphorus-containing groups | 74 of 971 in the list |
|  |  |
|  |  |
