# Supplementary material for: RNA-seq for comparative transcript profiling of kenaf under salinity stress
Source: J Plant Res. 2016 Dec 20;130(2):365–72. doi: 10.1007/s10265-016-0898-9 (PMC5318473; doi:10.1007/s10265-016-0898-9)
Supplement: Supplementary file 5 — Supplementary material 5 (DOCX 21 KB) [file 10265_2016_898_MOESM5_ESM.docx]

| **Table S5** KEGG metabolic pathways | | |
| --- | --- | --- |
| Pathway | Number of gene | Pathway ID |
| Metabolic pathways | 501 | ko01100 |
| Biosynthesis of secondary metabolites | 302 | ko01110 |
| Photosynthesis | 46 | ko00195 |
| Photosynthesis - antenna proteins | 19 | ko00196 |
| Glyoxylate and dicarboxylate metabolism | 41 | ko00630 |
| Phenylpropanoid biosynthesis | 57 | ko00940 |
| Flavone and flavonol biosynthesis | 38 | ko00944 |
| Carbon fixation in photosynthetic organisms | 40 | ko00710 |
| Nitrogen metabolism | 28 | ko00910 |
| Cutin, suberine and wax biosynthesis | 21 | ko00073 |
| Flavonoid biosynthesis | 38 | ko00941 |
| Carotenoid biosynthesis | 26 | ko00906 |
| Stilbenoid, diarylheptanoid and gingerol biosynthesis | 32 | ko00945 |
| Glycolysis / Gluconeogenesis | 42 | ko00010 |
| Zeatin biosynthesis | 27 | ko00908 |
| Cyanoamino acid metabolism | 20 | ko00460 |
| Diterpenoid biosynthesis | 18 | ko00904 |
| Glycine, serine and threonine metabolism | 24 | ko00260 |
| Limonene and pinene degradation | 24 | ko00903 |
| Fructose and mannose metabolism | 22 | ko00051 |
| Ether lipid metabolism | 39 | ko00565 |
| Pentose phosphate pathway | 20 | ko00030 |
| Alanine, aspartate and glutamate metabolism | 17 | ko00250 |
| Other glycan degradation | 16 | ko00511 |
| Starch and sucrose metabolism | 48 | ko00500 |
| Galactose metabolism | 18 | ko00052 |
| Glycerophospholipid metabolism | 49 | ko00564 |
| Pyruvate metabolism | 25 | ko00620 |
| Indole alkaloid biosynthesis | 6 | ko00901 |
| Valine, leucine and isoleucine biosynthesis | 11 | ko00290 |
| Phenylalanine metabolism | 14 | ko00360 |
| Arginine and proline metabolism | 19 | ko00330 |
| Thiamine metabolism | 4 | ko00730 |
| Pentose and glucuronate interconversions | 23 | ko00040 |
| Sesquiterpenoid and triterpenoid biosynthesis | 9 | ko00909 |
| Glutathione metabolism | 16 | ko00480 |
| Isoflavonoid biosynthesis | 5 | ko00943 |
| Monoterpenoid biosynthesis | 3 | ko00902 |
| Riboflavin metabolism | 7 | ko00740 |
| alpha-Linolenic acid metabolism | 12 | ko00592 |
| Porphyrin and chlorophyll metabolism | 12 | ko00860 |
| Peroxisome | 17 | ko04146 |
| Brassinosteroid biosynthesis | 5 | ko00905 |
| Linoleic acid metabolism | 5 | ko00591 |
| One carbon pool by folate | 5 | ko00670 |
| Citrate cycle (TCA cycle) | 13 | ko00020 |
| Cysteine and methionine metabolism | 14 | ko00270 |
| Biosynthesis of unsaturated fatty acids | 8 | ko01040 |
| Sulfur metabolism | 7 | ko00920 |
| Valine, leucine and isoleucine degradation | 9 | ko00280 |
| Glycosphingolipid biosynthesis - ganglio series | 4 | ko00604 |
| Fatty acid biosynthesis | 9 | ko00061 |
| Endocytosis | 45 | ko04144 |
| Sphingolipid metabolism | 7 | ko00600 |
| Propanoate metabolism | 8 | ko00640 |
| Synthesis and degradation of ketone bodies | 2 | ko00072 |
| Tryptophan metabolism | 8 | ko00380 |
| Fatty acid metabolism | 9 | ko00071 |
| Butanoate metabolism | 6 | ko00650 |
| Glucosinolate biosynthesis | 3 | ko00966 |
| Glycosaminoglycan degradation | 4 | ko00531 |
| Steroid biosynthesis | 7 | ko00100 |
| Sulfur relay system | 2 | ko04122 |
| Tropane, piperidine and pyridine alkaloid biosynthesis | 3 | ko00960 |
| Benzoxazinoid biosynthesis | 4 | ko00402 |
| Vitamin B6 metabolism | 3 | ko00750 |
| Ubiquinone and other terpenoid-quinone biosynthesis | 5 | ko00130 |
| Isoquinoline alkaloid biosynthesis | 3 | ko00950 |
| Ascorbate and aldarate metabolism | 10 | ko00053 |
| ABC transporters | 16 | ko02010 |
| Glycosylphosphatidylinositol(GPI)-anchor biosynthesis | 8 | ko00563 |
| Selenocompound metabolism | 4 | ko00450 |
| Terpenoid backbone biosynthesis | 10 | ko00900 |
| Anthocyanin biosynthesis | 1 | ko00942 |
| Arachidonic acid metabolism | 2 | ko00590 |
| Protein processing in endoplasmic reticulum | 44 | ko04141 |
| Glycerolipid metabolism | 10 | ko00561 |
| beta-Alanine metabolism | 5 | ko00410 |
| C5-Branched dibasic acid metabolism | 1 | ko00660 |
| Homologous recombination | 7 | ko03440 |
| Pyrimidine metabolism | 25 | ko00240 |
| Histidine metabolism | 3 | ko00340 |
| Amino sugar and nucleotide sugar metabolism | 16 | ko00520 |
| Pantothenate and CoA biosynthesis | 3 | ko00770 |
| Purine metabolism | 24 | ko00230 |
| Oxidative phosphorylation | 15 | ko00190 |
| Base excision repair | 4 | ko03410 |
| Tyrosine metabolism | 4 | ko00350 |
| RNA polymerase | 11 | ko03020 |
| Non-homologous end-joining | 1 | ko03450 |
| Natural killer cell mediated cytotoxicity | 9 | ko04650 |
| Other types of O-glycan biosynthesis | 1 | ko00514 |
| Phenylalanine, tyrosine and tryptophan biosynthesis | 3 | ko00400 |
| Lysine degradation | 4 | ko00310 |
| Fatty acid elongation | 2 | ko00062 |
| Circadian rhythm - plant | 20 | ko04712 |
| Nucleotide excision repair | 6 | ko03420 |
| Plant-pathogen interaction | 68 | ko04626 |
| Plant hormone signal transduction | 90 | ko04075 |
| Aminoacyl-tRNA biosynthesis | 4 | ko00970 |
| Proteasome | 4 | ko03050 |
| Phagosome | 10 | ko04145 |
| Mismatch repair | 2 | ko03430 |
| Ribosome biogenesis in eukaryotes | 15 | ko03008 |
| Protein export | 2 | ko03060 |
| Inositol phosphate metabolism | 5 | ko00562 |
| Regulation of autophagy | 4 | ko04140 |
| DNA replication | 1 | ko03030 |
| Phosphatidylinositol signaling system | 5 | ko04070 |
| SNARE interactions in vesicular transport | 1 | ko04130 |
| RNA transport | 42 | ko03013 |
| Basal transcription factors | 3 | ko03022 |
| Ubiquitin mediated proteolysis | 16 | ko04120 |
| Spliceosome | 27 | ko03040 |
| RNA degradation | 9 | ko03018 |
| mRNA surveillance pathway | 13 | ko03015 |
| Ribosome | 14 | ko03010 |
|  |  |  |
