# Supplementary material for: RNA-seq for comparative transcript profiling of kenaf under salinity stress
Source: J Plant Res. 2016 Dec 20;130(2):365–72. doi: 10.1007/s10265-016-0898-9 (PMC5318473; doi:10.1007/s10265-016-0898-9)
Supplement: Supplementary file 6 — Supplementary material 6 (DOCX 18 KB) [file 10265_2016_898_MOESM6_ESM.docx]

| **Table S6** Free amino acid and soluble sugar matabolism pathways | | | |
| --- | --- | --- | --- |
| Pathway | Number of genes | Pathway ID | Genes |
| Glycine, serine and threonine metabolism | 23 | ko00260 | Unigene3318_All; CL4683.Contig1_All; CL4683.Contig4_All; CL4683.Contig5_All; CL4683.Contig6_All; Unigene26368_All; CL7715.Contig2_All; CL7715.Contig1_All; CL7240.Contig2_All; CL7623.Contig2_All; Unigene876_All; CL7715.Contig3_All; CL6269.Contig2_All; CL4987.Contig3_All; CL9049.Contig2_All; Unigene28183_All; CL8985.Contig2_All; CL4987.Contig4_All; CL4683.Contig2_All; Unigene9352_All; CL7440.Contig1_All; CL4987.Contig2_All; CL6876.Contig1_All |
| Fructose and mannose metabolism | 18 | ko00051 | CL754.Contig7_All; CL3166.Contig2_All; CL754.Contig8_All; Unigene13166_All; CL3944.Contig4_All; CL3944.Contig5_All; CL3944.Contig1_All; CL754.Contig3_All; CL6440.Contig2_All; CL754.Contig2_All; Unigene11569_All; Unigene6558_All; Unigene30322_All; CL8215.Contig2_All; CL2942.Contig1_All; Unigene12482_All; CL4158.Contig1_All; CL4534.Contig2_All |
| Phenylalanine metabolism | 14 | ko00360 | CL2150.Contig1_All; Unigene828_All; Unigene9430_All; CL2984.Contig2_All; Unigene3456_All; Unigene4696_All; Unigene18048_All; CL3619.Contig2_All; CL5798.Contig4_All; CL9498.Contig1_All; Unigene6716_All; Unigene9405_All; Unigene6569_All; Unigene9352_All |
| Valine, leucine and isoleucine biosyntheses | 10 | ko00290 | Unigene3318_All; Unigene2097_All; Unigene27486_All; CL9559.Contig1_All; CL6497.Contig1_All; Unigene876_All; CL3987.Contig3_All; Unigene14885_All; CL3987.Contig1_All; CL8033.Contig2_All |
| Galactose metabolism | 14 | ko00052 | Unigene32927_All; CL8127.Contig1_All; Unigene24910_All; CL3923.Contig1_All; CL6440.Contig2_All; CL2850.Contig1_All; Unigene11569_All; CL7937.Contig4_All; Unigene7196_All; Unigene12482_All; CL2850.Contig2_All; CL4534.Contig2_All; CL7937.Contig1_All; CL2204.Contig2_All |
| Starch and sucrose metabolism | 34 | ko00500 | Unigene32927_All; CL8127.Contig1_All; CL4528.Contig4_All; CL2390.Contig2_All; Unigene15082_All; Unigene3649_All; CL3422.Contig1_All; CL4036.Contig1_All; Unigene24910_All; CL9647.Contig1_All; CL4164.Contig3_All; Unigene9377_All; CL4515.Contig1_All; CL2927.Contig3_All; CL3201.Contig2_All; CL2598.Contig1_All; Unigene811_All; CL2927.Contig9_All; Unigene17393_All; Unigene18552_All; CL3372.Contig1_All; Unigene11895_All; Unigene8760_All; CL3372.Contig2_All; Unigene7196_All; Unigene15360_All; CL2598.Contig2_All; CL6712.Contig1_All; CL4640.Contig3_All; CL4164.Contig1_All; CL3201.Contig1_All; CL4528.Contig3_All; CL599.Contig1_All; CL2204.Contig2_All |
| Arginine and proline metabolism | 11 | ko00330 | CL5175.Contig3_All; CL5175.Contig4_All; CL4975.Contig3_All; CL5175.Contig2_All; CL5175.Contig1_All; Unigene800_All; CL1373.Contig4_All; Unigene22040_All; CL4185.Contig2_All; Unigene11086_All; CL4971.Contig1_All |
| Cysteine and methionine metabolism | 9 | ko00270 | Unigene9430_All; CL4721.Contig6_All; CL4721.Contig9_All; CL1201.Contig10_All; Unigene6722_All; Unigene11881_All; Unigene1338_All; CL9149.Contig1_All; Unigene15839_All |
| Phenylalanine, tyrosine and tryptophan biosynthesis | 3 | ko00400 | Unigene9430_All; Unigene26368_All; CL9639.Contig2_All |
