# Supplementary material for: RNA-seq for comparative transcript profiling of kenaf under salinity stress
Source: J Plant Res. 2016 Dec 20;130(2):365–72. doi: 10.1007/s10265-016-0898-9 (PMC5318473; doi:10.1007/s10265-016-0898-9)
Supplement: Supplementary file 8 — Supplementary material 8 (DOCX 19 KB) [file 10265_2016_898_MOESM8_ESM.docx]

| **Table S7** Transcription factors in response to salt stress | | | |
| --- | --- | --- | --- |
| geneID | log2 Ratio(ST/CK) | Up-Down-Regulation(ST/CK) | Nr-annotation |
| Unigene2610_All | 12.08500764 | Up | AP2/ERF domain-containing transcription factor |
| Unigene8081_All | 3.369976614 | Up | CCCH-type zinc finger transcription factor |
| Unigene17994_All | 2.960083768 | Up | transcription factor bHLH123 |
| CL3690.Contig1_All | 2.778277689 | Up | transcription factor bHLH113 |
| Unigene24983_All | 2.545024464 | Up | MADS-box protein |
| Unigene13879_All | 2.273655408 | Up | transcription factor TCP20 |
| CL1928.Contig2_All | 2.266501439 | Up | transcription factor bHLH70 |
| CL2760.Contig4_All | 2.248605979 | Up | transcription factor TCP20 |
| CL2614.Contig2_All | 2.115724579 | Up | transcription factor bHLH137 |
| CL635.Contig5_All | 1.897290265 | Up | trihelix transcription factor GT-2 |
| Unigene14881_All | 1.84615765 | Up | ethylene-responsive transcription factor 5 |
| Unigene1363_All | 1.755578402 | Up | transcription factor bHLH123 |
| Unigene9858_All | 1.701132127 | Up | ethylene-responsive transcription factor 5 |
| CL1246.Contig1_All | 1.611274983 | Up | ethylene-responsive element binding protein |
| Unigene23754_All | 1.526504191 | Up | NAC domain-containing protein, putative |
| CL7954.Contig1_All | 1.460132126 | Up | ethylene-responsive transcription factor |
| Unigene9216_All | 1.415740415 | Up | transcription factor TM6 |
| CL4743.Contig4_All | 1.37508905 | Up | Nuclear transcription factor Y subunit A-3 |
| CL2785.Contig4_All | 1.312610005 | Up | AP2/EREBP transcription factor ERF-1 |
| Unigene6542_All | 1.075967749 | Up | ARF domain class transcription factor |
| Unigene4519_All | 1.071047881 | Up | BZIP domain class transcription factor |
| CL1246.Contig2_All | –13.47996968 | Down | ethylene-responsive element binding protein |
| Unigene8137_All | –3.736116437 | Down | AP2/ERF domain-containing transcription factor |
| CL1984.Contig1_All | –2.025039757 | Down | NAC domain protein NAC5 |
| CL1984.Contig2_All | –1.906232428 | Down | NAC domain protein NAC5 |
| Unigene23699_All | –1.835837068 | Down | NAC domain protein NAC4 |
| CL2314.Contig4_All | –1.742562634 | Down | NAC domain protein, IPR003441 |
| Unigene11994_All | –1.550744778 | Down | AP2/EREBP transcription factor ERF-1 |
| CL1984.Contig4_All | –1.368122282 | Down | NAC protein 8 |
| Unigene14730_All | –1.326706248 | Down | WRKY transcription factor |
| CL6333.Contig1_All | –1.308501016 | Down | WRKY transcription factor |
| Unigene17321_All | –1.218645749 | Down | AP2/ERF domain-containing transcription factor |
| CL3430.Contig3_All | –1.157585046 | Down | NAC domain-containing protein |
| Unigene6347_All | –1.157472562 | Down | zinc finger protein |
| CL3430.Contig2_All | –1.028726427 | Down | NAC domain-containing protein |
| CL6531.Contig1_All | –1.011446222 | Down | NAC domain protein NAC2 |
|  |  |  |  |
